# Supplementary figures and images for: Anti-Migraine Effect of the Herbal Combination of Chuanxiong Rhizoma and Cyperi Rhizoma and UPLC-MS/MS Method for the Simultaneous Quantification of the Active Constituents in Rat Serum and Cerebral Cortex
Source: Molecules. 2019 Jun 14;24(12):2230. doi: 10.3390/molecules24122230 (PMC6630925; doi:10.3390/molecules24122230)

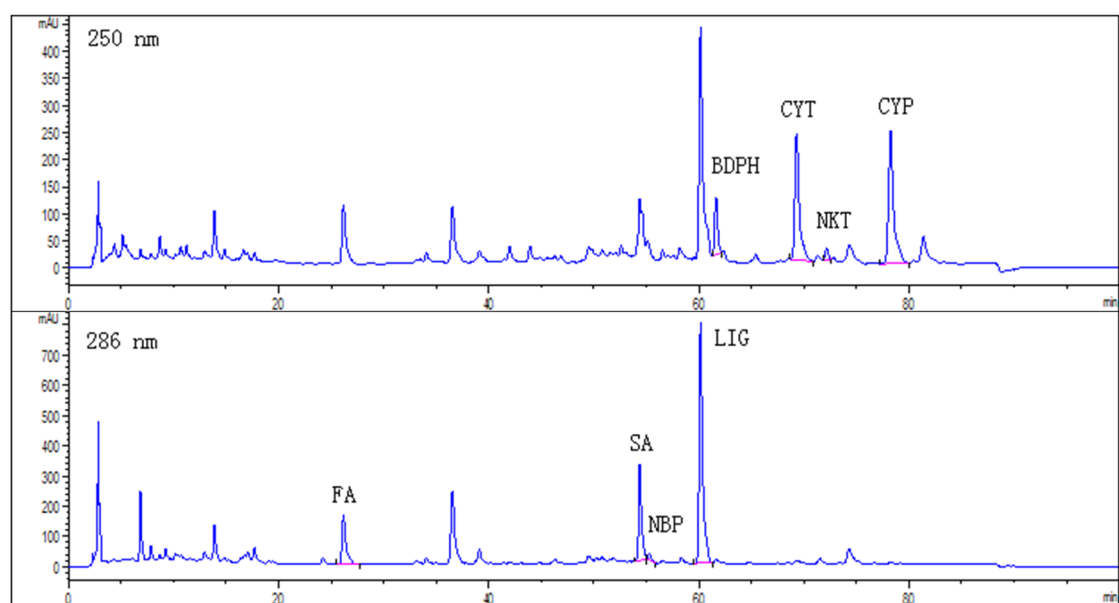

Figure S1. The HPLC chromatograms of CRCR extract solution.

Supplement: Supplementary file 1 [file molecules-24-02230-s001.pdf]
